# Supplementary material for: Strong linkage between benthic oxygen uptake and bacterial tetraether lipids in deep-sea trench regions
Source: Nat Commun. 2024 Apr 23;15:3439. doi: 10.1038/s41467-024-47660-3 (PMC11039702; doi:10.1038/s41467-024-47660-3)
Supplement: Supplementary file 1 — Supplementary Information [file 41467_2024_47660_MOESM1_ESM.pdf]

Supporting Information for

Strong linkage between benthic oxygen uptake and bacterial tetraether lipids in deep-sea trench regions

Wenjie Xiao[1,2,3*], Yunping Xu[2*], Donald E. Canfield[1,4], Frank Wenzhöfer[1,5,6], Chuanlun Zhang[3,7], Ronnie N. Glud[1,2,4,8*]

[1]Department of Biology, HADAL & Nordcee, University of Southern Denmark, 5230 Odense M, Denmark

[2]Shanghai Frontiers Research Center of the Hadal Biosphere, College of Oceanography and Ecological Science, Shanghai Ocean University, 201306 Shanghai, China

[3]Shenzhen Key Laboratory of Marine Archaea Geo-Omics, Department of Ocean Science and Engineering, Southern University of Science and Technology, 518055 Shenzhen, China

[4]Danish Institute for Advanced Study (DIAS), University of Southern Denmark, 5230 Odense M, Denmark.

[5]HGF-MPG Group for Deep Sea Ecology & Technology, Alfred Wegener Institute Helmholtz Centre for Polar- and Marine Research, Am Handelshafen 12, 27570 Bremerhaven, Germany

[6]Max Planck Institute for Marine Microbiology, Celsiusstr 1, D-28359 Bremen, Germany

[7]Shanghai Sheshan National Geophysical Observatory, 201602 Shanghai, China

[8]Department of Ocean and Environmental Sciences, Tokyo University of Marine Science and Technology, 26 108-8477 Tokyo, Japan

***Corresponding author:** Wenjie Xiao (wjxiaocug@126.com); Yunping Xu (ypxu@shou.edu.cn); Ronnie N. Glud (rnglud@biology.sdu.dk)

This PDF file includes:

Figures S1 to S6

Table S1

SI References

Other supporting materials for this manuscript include the following:

Supplementary Data 1

Supplementary Data 2

Supplementary Data 3

Supplementary Data 4

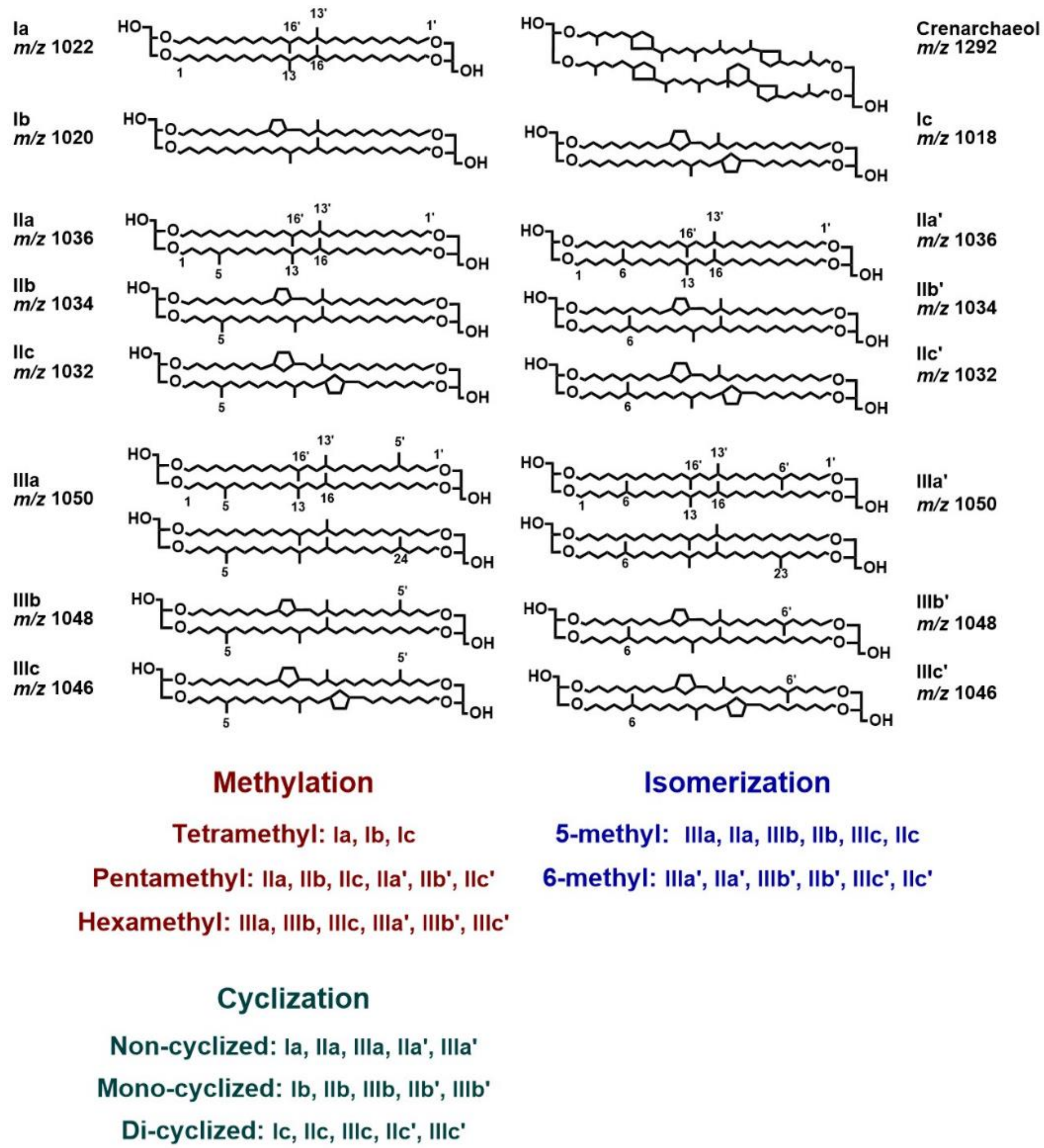

Figure S1. Molecular structures of branched glycerol dialkyl glycerol tetraethers (brGDGTs) and crenarchaeol, along with a schematic of the brGDGT structure sets. The methylation degree of brGDGTs can be categorized into tetramethylated, pentamethylated, and hexamethylated compounds. The isomerization degree of brGDGTs can be categorized into 5-methyl and 6-methyl compounds. The cyclization degree of brGDGTs can be categorized into non-cyclized, mono-cyclized and di-cyclized compounds. These classifications provide a systematic understanding of the structural variations within the brGDGT molecule.

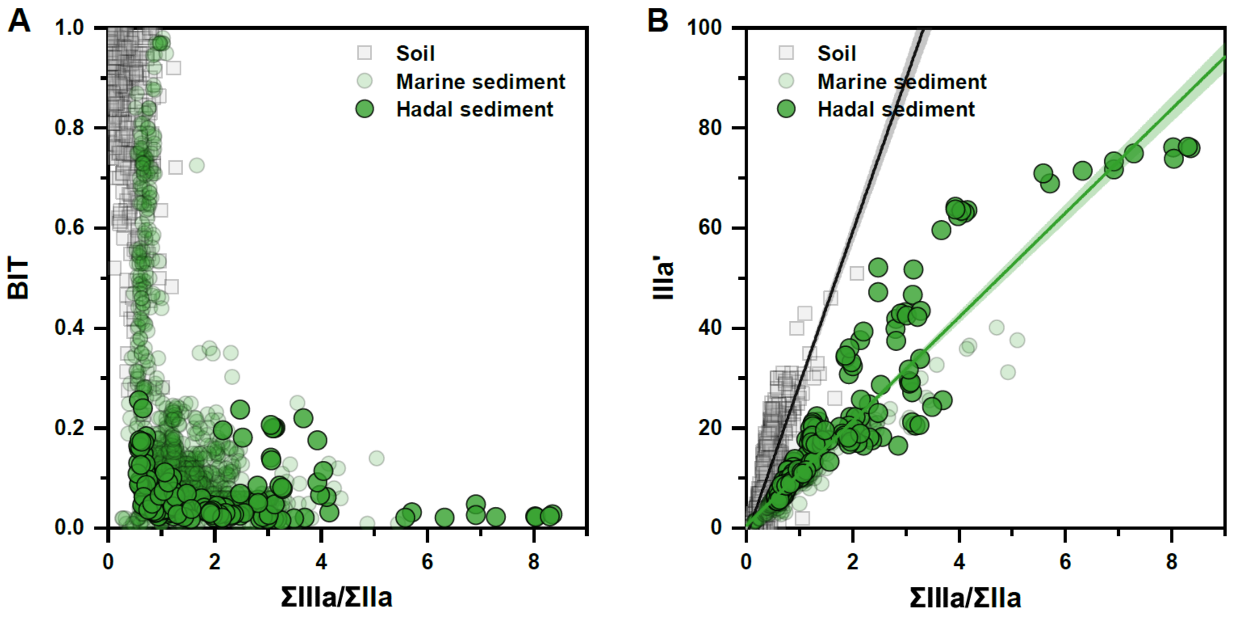

Figure S2. In situ production of branched glycerol dialkyl glycerol tetraethers (brGDGTs) in hadal sediments. A Scatterplot showing the ΣIIIa/ΣIIa index and BIT index in hadal sediments (dark green circles) from this study, alongside global soils (gray squares) and marine sediments (light green circles) obtained from Xiao et al. (2016)[1], which compiled data from various literature sources. The ΣIIIa/ΣIIa index serves as a valuable tool for assessing the origin of brGDGTs in marine environments, which typically yields values < 0.59 in soils and between 0.59 – 0.92 and > 0.92 in marine sediments with and without terrestrial influence, respectively. **B** Scatterplot comparing the ΣIIIa/ΣIIa index and the fractional abundance of IIIa' compound. Linear regression lines are depicted in black for soils and green for marine sediments (including hadal sediments), along with 95% confidence intervals shown as gray bands for soils and green bands for marine sediments. The soil and marine sediment data are sourced from Xiao et al. (2020)[2], which integrates data from various literature sources. The slope of the regression line aids in deciphering the terrestrial and marine provenance of brGDGTs, with the former exhibiting higher values than the latter.

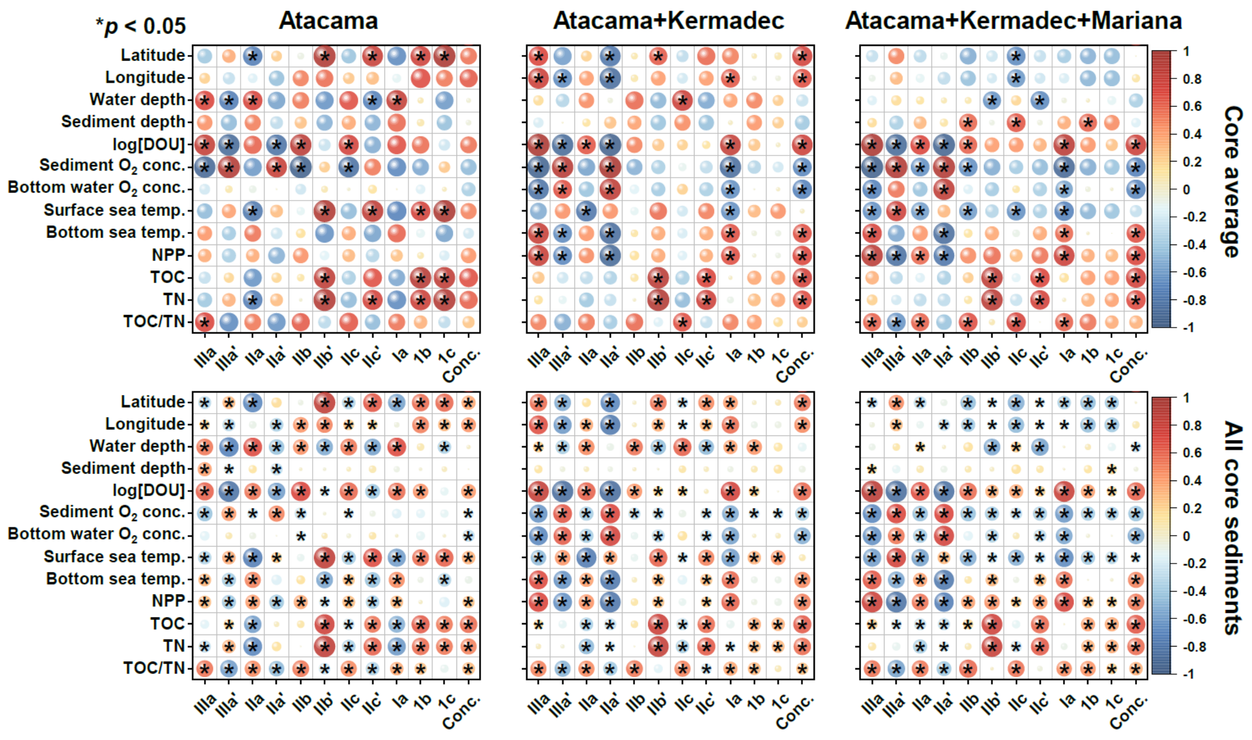

Figure S3. Correlation maps between branched glycerol dialkyl glycerol tetraethers (brGDGTs) and environmental variables. Pearson correlation analysis was utilized to assess the relationships between brGDGTs, both in terms of fractional abundances and concentrations (conc.), and a range of environmental variables. These variables include latitude, longitude, water depth, sediment depth, diffusive oxygen uptake (DOU), dissolved oxygen concentrations in bottom water and sediment, annual sea surface and bottom temperatures, net primary productivity (NPP), total organic carbon (TOC), total nitrogen (TN), and TOC/TN ratio (Supplementary Data 3). Notice a logarithmic scale of DOU. The colormap indicates the correlation coefficients (R values), with red indicating positive correlations and blue indicating negative correlations. The *p* values are derived from two-sided Student's t-tests, with $*p < 0.05$ indicating significance. Due to the limited number of cores from the Kermadec Trench (n = 4) and the Mariana Trench (n = 1), conducting separate correlation analyses for these trenches was impractical. Alternatively, we performed correlation analyses on samples from the Atacama trench, combined samples from the Atacama and Kermadec trenches, and combined samples from all the trenches. Analyses were performed individually for the average values of each core and for the entire core sample dataset, while analyses for surface samples are presented in Fig. 4 of the main text. Regardless of whether the analysis focused on a single trench or all trenches

combined, or the comparison of surface sediments with all core sediments, DOU emerged as the strongest and most significant parameter influencing brGDGTs. Note that DOU primarily affects brGDGT compounds that reflect the methylation and isomerization degrees, which constitute more than 70% of the total brGDGTs.

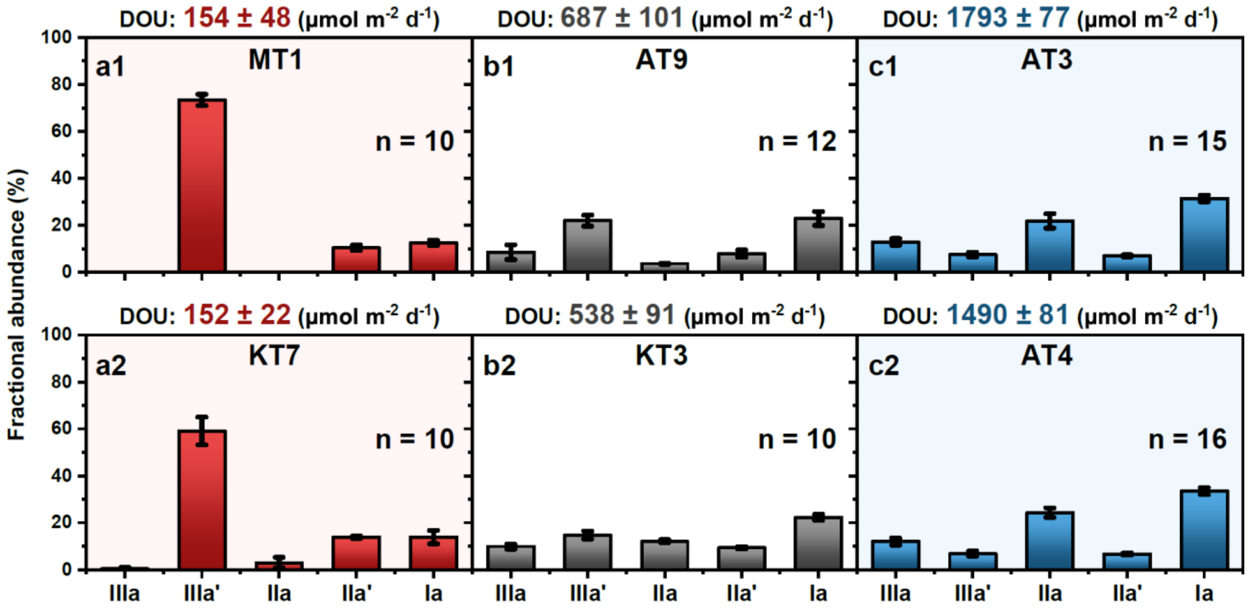

Figure S4. Branched glycerol dialkyl glycerol tetraethers (brGDGTs) compositions vary under different diffusive oxygen uptake (DOU) conditions. Sites with low DOU, such as **a1** MT1 and **a2** KT7, are primarily composed of IIIa' compound. Moderate DOU sites like **b1** AT9 and **b2** KT3 exhibit a notable presence of both IIIa' and Ia compounds as the main components. Sites with high DOU levels, such as **c1** AT3 and **c2** AT4, are mainly characterized by the presence of Ia and IIa compounds. The columns with error bars denote mean values with standard deviations of variables for each sediment core, with sample sizes indicated within the plot. Note that only Ia, IIa, IIIa, IIa', and IIIa' compounds that primarily reflect the degree of methylation and isomerization of brGDGTs are shown.

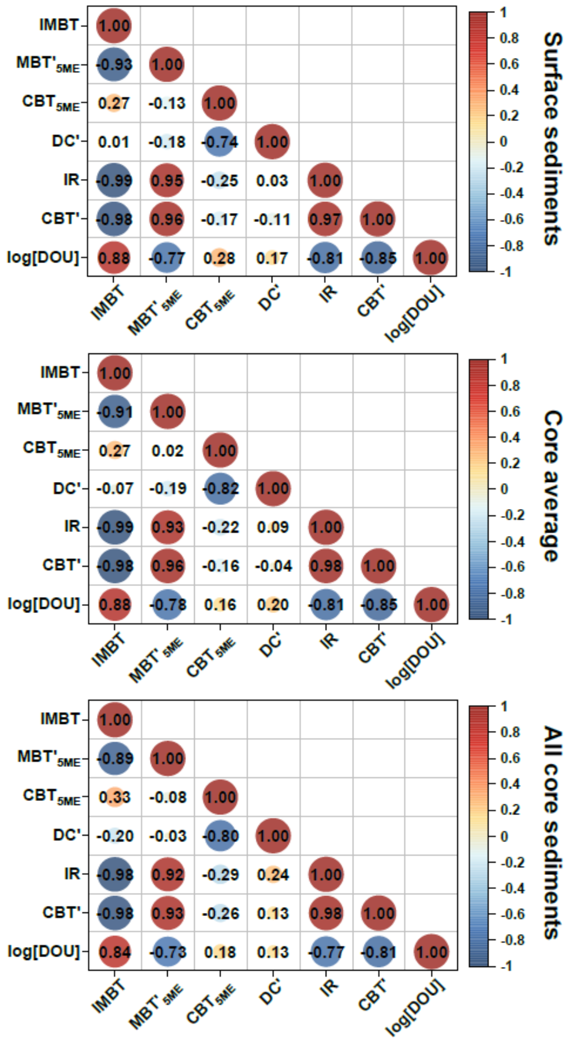

Figure S5. Correlation maps between branched glycerol dialkyl glycerol tetraethers (brGDGTs) indexes and diffusive oxygen uptake (DOU). Pearson correlation analysis was employed to assess the relationships between multiple brGDGT indexes and DOU. The examined indexes include IMBT, MBT'$_{5ME}$, CBT$_{5ME}$, DC', IR, and CBT'. The calculations for DC' and CBT' were based on equations from De Jonge et al. (2021)[3] and De Jonge et al. (2014)[4], respectively. Descriptions of the other indexes are provided in the Methods section of the main text. Notice a logarithmic scale of DOU. The colormap indicates the correlation coefficients (R values), with red indicating positive correlations and blue indicating negative correlations. Correlation analyses were performed individually for surface samples, the average values of each core, and the entire core sample dataset. Notably, IMBT, MBT'$_{5ME}$, IR, and CBT' show strong correlations with DOU, while CBT$_{5ME}$ and DC' exhibit much weaker correlations.

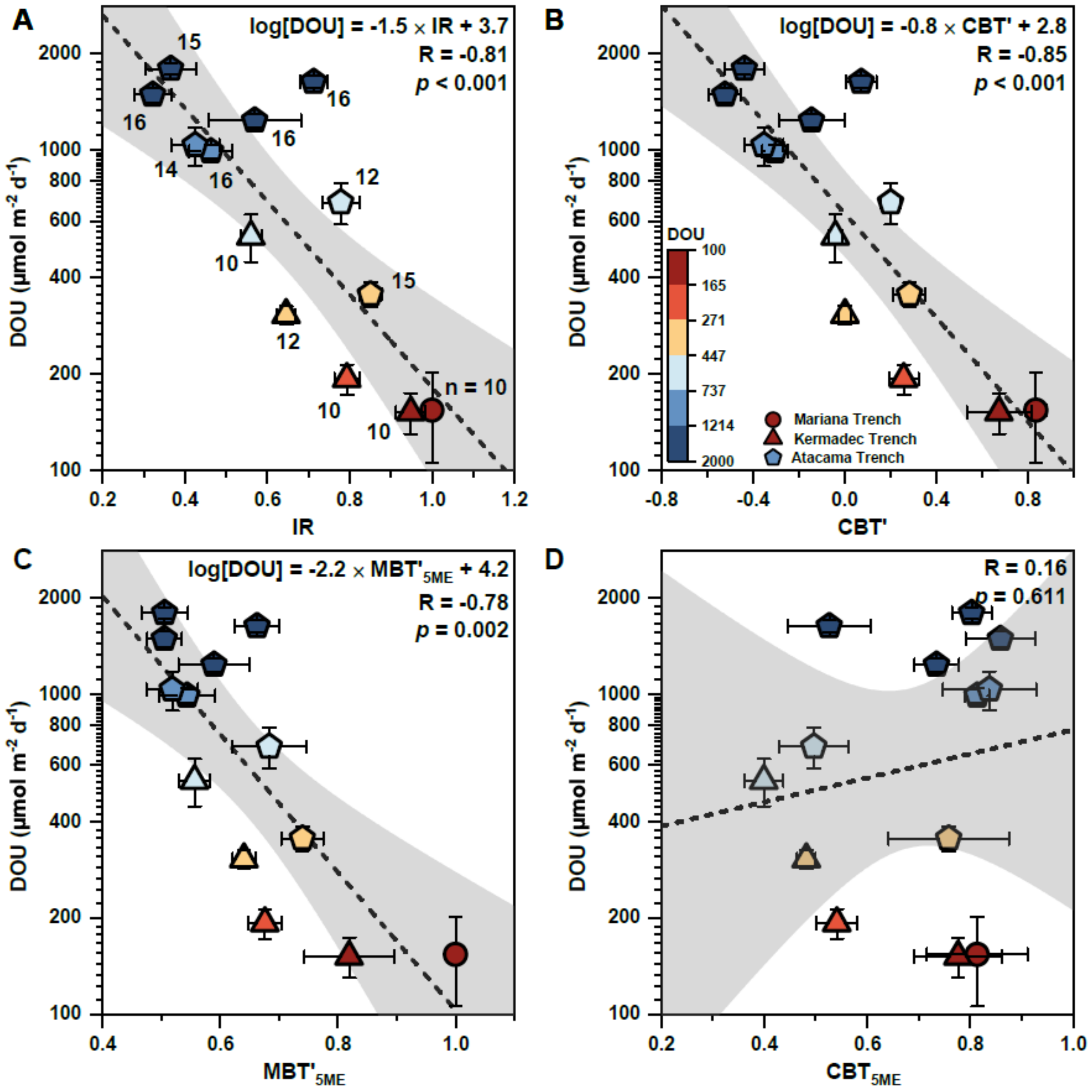

Figure S6. Correlation between diffusive oxygen uptake (DOU) and commonly used branched glycerol dialkyl glycerol tetraethers (brGDGTs)-based indexes. Scatterplot comparing the **A** IR, **B** CBT', **C** MBT'$_{5ME}$, and **D** CBT$_{5ME}$ indexes with DOU. Notice a logarithmic scale of DOU. Linear regression line (black) and 95% confidence intervals (gray band) are shown. Pearson correlation coefficients (R values) are provided for each plot. All reported *p* values result from two-sided Student's t-test. The circle, triangle and pentagon symbols with error bars denote mean values with standard deviations of variables for sediment cores from the Mariana, Kermadec, and Atacama trench regions, respectively, with sample sizes provided within the plot. The colormap indicates the DOU values of the hadal samples.

Table S1. Summary of main data presented in the Results and Discussion section of the main text. Main data including sedimentation rates, diffusive oxygen uptake (DOU) values, concentrations and fractional abundances of branched glycerol dialkyl glycerol tetraethers (brGDGTs), along with related brGDGT proxies, from sites in the Mariana Trench (MT), Kermadec Trench (KT) and Atacama Trench (AT) regions. Sedimentation rate data are cited from Glud et al. (2013)[5], Oguri et al. (2022)[6], and Zabel et al. (2022)[7]. DOU data are cited from Glud et al. (2013)[5] and Glud et al. (2021)[8]. Data on brGDGT concentrations, fractional abundances, and related proxies were generated in this study, which have been detailed in the Supplementary Data 2.

Parameter	MT site (n = 1)	KT sites (n = 4)	AT sites (n = 8)
Sedimentation rate (cm yr^{-1})	0.04	0.03 – 0.04	0.03 – 0.08
DOU (μmol m^{-2} d^{-1})	154	152 – 538	355 – 1793
BrGDGT concentration (ng g^{-1})	15±3	12±7	215±170
BrGDGT concentration normalized by TOC (μg g^{-1}TOC)	5±1	4±1	36±29
BrGDGT-IIIa (%)	0±0	5±3	10±3
BrGDGT-IIIa' (%)	73±2	32±17	15±9
BrGDGT-IIa (%)	0±0	8±4	14±8
BrGDGT-IIa' (%)	10±1	11±2	8±1
BrGDGT-IIb (%)	0±0	3±2	2±1
BrGDGT-IIb' (%)	1±0	6±2	9±5
BrGDGT-IIc (%)	0±0	1±0	1±0
BrGDGT-IIc' (%)	0±0	2±1	3±2
BrGDGT-Ia (%)	12±1	21±5	29±4
BrGDGT-Ib (%)	2±0	6±3	5±1
BrGDGT-Ic (%)	1±0	3±1	3±1
Non-cyclized brGDGTs (%)	96±1	78±8	76±7

Mono-cyclized brGDGTs (%)	2±1	16±6	17±5
Di-cyclized brGDGTs (%)	1±0	6±2	7±2
6-methyl brGDGTs (%)	100±0	73±15	56±19
5-methyl brGDGTs (%)	0±0	27±15	44±19
Hexamethylated brGDGTs (%)	73±2	37±14	26±7
Pentamethylated brGDGTs (%)	11±1	32±6	38±4
Tetramethylated brGDGTs (%)	15±2	31±8	37±4
BIT	0.03±0.01	0.07±0.05	0.07±0.05
ΣIIIa/ΣIIa	7.14±0.98	2.00±1.03	1.45±0.91
IR	1.00±0.00	0.73±0.15	0.56±0.19
CBT'	0.84±0.04	0.21±0.29	-0.16±0.29
MBT'_{5ME}	1.00±0.00	0.67±0.10	0.59±0.10
CBT_{5ME}	0.81±0.10	0.55±0.15	0.73±0.15
DC'	0.10±0.02	0.27±0.06	0.26±0.10
IMBT	0.06±0.01	0.30±0.14	0.55±0.19

SI References

1. Xiao W, Wang Y, Zhou S, Hu L, Yang H, Xu Y. Ubiquitous production of branched glycerol dialkyl glycerol tetraethers (brGDGTs) in global marine environments: A new source indicator for brGDGTs. *Biogeosciences* **13**, 5883–5894 (2016).

2. Xiao W, Wang Y, Liu Y, Zhang X, Shi L, Xu Y. Predominance of hexamethylated 6-methyl branched glycerol dialkyl glycerol tetraethers in the Mariana Trench: source and environmental implication. *Biogeosciences* **17**, 2135–2148 (2020).

3. De Jonge C, Kuramae EE, Radujković D, Weedon JT, Janssens IA, Peterse F. The influence of soil chemistry on branched tetraether lipids in mid- and high latitude soils: Implications for brGDGT- based paleothermometry. *Geochim. Cosmochim. Acta* **310**, 95–112 (2021).

4. De Jonge C, Hopmans EC, Zell CI, Kim J-H, Schouten S, Sinninghe Damsté JS. Occurrence and abundance of 6-methyl branched glycerol dialkyl glycerol tetraethers in soils: Implications for palaeoclimate reconstruction. *Geochim. Cosmochim. Acta* **141**, 97–112 (2014).

5. Glud RN*, et al.* High rates of microbial carbon turnover in sediments in the deepest oceanic trench on Earth. *Nat. Geosci.* **6**, 284–288 (2013).

6. Oguri K*, et al.* Sediment Accumulation and Carbon Burial in Four Hadal Trench Systems. *J. Geophys. Res.: Biogeosci.*, e2022JG006814 (2022).

7. Zabel M*, et al.* High carbon mineralization rates in subseafloor hadal sediments - Result of frequent mass wasting. *Geochem. Geophys. Geosyst.*, e2022GC010502 (2022).

8. Glud RN*, et al.* Hadal trenches are dynamic hotspots for early diagenesis in the deep sea. *Commun. Earth Environ.* **2**, 21 (2021).
