# Supplementary material for: Strong linkage between benthic oxygen uptake and bacterial tetraether lipids in deep-sea trench regions
Source: Nat Commun. 2024 Apr 23;15:3439. doi: 10.1038/s41467-024-47660-3 (PMC11039702; doi:10.1038/s41467-024-47660-3)
Supplement: Supplementary file 3 — Description of Additional Supplementary Files [file 41467_2024_47660_MOESM3_ESM.pdf]

Description of Additional Supplementary Files

File Name: Supplementary Data 1
Description: Locations, water depths, sediment depths, and core types at the investigated sites in the Kermadec, Atacama, and Mariana trench regions.

File Name: Supplementary Data 2
Description: Fractional abundance and concentration of branched glycerol dialkyl glycerol tetraethers (brGDGTs), along with brGDGT-based proxies and their reconstructed variables in the core sediments from the Kermadec, Atacama, and Mariana trench regions.

File Name: Supplementary Data 3
Description: Environmental variables, including location, water depth, sediment depth, annual sea surface and bottom temperature, bottom water and sediment dissolved oxygen concentrations, diffusive oxygen uptake (DOU), total organic carbon (TOC), total nitrogen (TN), the TOC/TN ratio, and net primary productivity (NPP) in the core sediments from the Kermadec, Atacama, and Mariana trench regions. The data are obtained from various studies, and have been explained in the Data Compilation section under Methods.

File Name: Supplementary Data 4
Description: Fractional abundance of branched glycerol dialkyl glycerol tetraethers (brGDGTs) and derived Methylation index of Branched Tetraethers (IMBT) compiled from the literature. The reference for the data have been listed in the table and their sources have also been explained in the Data Compilation section under Methods.
