# Supplementary material for: Strong linkage between benthic oxygen uptake and bacterial tetraether lipids in deep-sea trench regions
Source: Nat Commun. 2024 Apr 23;15:3439. doi: 10.1038/s41467-024-47660-3 (PMC11039702; doi:10.1038/s41467-024-47660-3)
Supplement: Supplementary file 8 — Reporting Summary [file 41467_2024_47660_MOESM8_ESM.pdf]

nature portfolio

Corresponding author(s): Wenjie Xiao

Last updated by author(s): Apr 1, 2024

Reporting Summary

Nature Portfolio wishes to improve the reproducibility of the work that we publish. This form provides structure for consistency and transparency in reporting. For further information on Nature Portfolio policies, see our Editorial Policies and the Editorial Policy Checklist.

Statistics

For all statistical analyses, confirm that the following items are present in the figure legend, table legend, main text, or Methods section.

n/a	Confirmed	
☐	☒	The exact sample size (*n*) for each experimental group/condition, given as a discrete number and unit of measurement
☐	☒	A statement on whether measurements were taken from distinct samples or whether the same sample was measured repeatedly
☐	☒	The statistical test(s) used AND whether they are one- or two-sided<br>*Only common tests should be described solely by name; describe more complex techniques in the Methods section.*
☒	☐	A description of all covariates tested
☐	☒	A description of any assumptions or corrections, such as tests of normality and adjustment for multiple comparisons
☐	☒	A full description of the statistical parameters including central tendency (e.g. means) or other basic estimates (e.g. regression coefficient) AND variation (e.g. standard deviation) or associated estimates of uncertainty (e.g. confidence intervals)
☐	☒	For null hypothesis testing, the test statistic (e.g. *F*, *t*, *r*) with confidence intervals, effect sizes, degrees of freedom and *P* value noted<br>*Give P values as exact values whenever suitable.*
☒	☐	For Bayesian analysis, information on the choice of priors and Markov chain Monte Carlo settings
☒	☐	For hierarchical and complex designs, identification of the appropriate level for tests and full reporting of outcomes
☐	☒	Estimates of effect sizes (e.g. Cohen's *d*, Pearson's *r*), indicating how they were calculated

Our web collection on statistics for biologists contains articles on many of the points above.

Software and code

Policy information about availability of computer code

Data collection	Branched glycerol dialkyl glycerol tetraethers (brGDGTs) were analyzed using an Agilent 1260 series High-Performance Liquid Chromatography (HPLC) system coupled with an Agilent 6135B quadrupole Mass Spectrometer (MS) with an Atmospheric Pressure Chemical Ionization (APCI) source. The integration of brGDGT compound peaks was performed with Agilent MassHunter Qualitative Analysis software (version 10.0).
Data analysis	The maps in this study were created utilizing Ocean Data View (ODV version 5.6.3) and ArcMap version 10.7 software. Principal Coordinates Analysis (PCoA) was conducted using the vegan package in R version 4.2.1, with the Bray Curtis method employed to generate the distance matrix. To investigate variations in brGDGT compositions and related indexes across different trench sites, one-way analysis of variance (one-way ANOVA) was performed using SPSS version 22.0 software. The molecular structures of brGDGTs were generated using ChemDraw version 19.0 software, while other graphical representations were generated using OriginPro version 9.8.0.200 software.

For manuscripts utilizing custom algorithms or software that are central to the research but not yet described in published literature, software must be made available to editors and reviewers. We strongly encourage code deposition in a community repository (e.g. GitHub). See the Nature Portfolio guidelines for submitting code & software for further information.

Data

Policy information about availability of data

All manuscripts must include a data availability statement. This statement should provide the following information, where applicable:

- Accession codes, unique identifiers, or web links for publicly available datasets
- A description of any restrictions on data availability
- For clinical datasets or third party data, please ensure that the statement adheres to our policy

All the data supporting the findings from this study are provided in this paper and its Supplementary Information. The WOA18 0.25 degree dataset is available at https://www.ncei.noaa.gov/products/world-ocean-atlas. The net primary productivity (NPP) dataset, based on the standard Vertically Generalized Production Model, is available at http://orca.science.oregonstate.edu/1080.by.2160.monthly.hdf.vgpm.m.chl.m.sst.php.

Research involving human participants, their data, or biological material

Policy information about studies with human participants or human data. See also policy information about sex, gender (identity/presentation), and sexual orientation and race, ethnicity and racism.

Reporting on sex and gender	N/A
Reporting on race, ethnicity, or other socially relevant groupings	N/A
Population characteristics	N/A
Recruitment	N/A
Ethics oversight	N/A

Note that full information on the approval of the study protocol must also be provided in the manuscript.

Field-specific reporting

Please select the one below that is the best fit for your research. If you are not sure, read the appropriate sections before making your selection.

☐ Life sciences ☐ Behavioural & social sciences ☒ Ecological, evolutionary & environmental sciences

For a reference copy of the document with all sections, see nature.com/documents/nr-reporting-summary-flat.pdf

Ecological, evolutionary & environmental sciences study design

All studies must disclose on these points even when the disclosure is negative.

Study description	This study investigates the relationship between oxygen conditions in marine sediments and branched glycerol dialkyl glycerol tetraethers (brGDGTs) produced by marine bacteria. By examining brGDGTs in 13 sediment cores from three deep-sea trench regions, at depths of 4,045 to 10,100 meters, and integrating this with oxygen microprofile data, it finds a strong linkage between brGDGT distributions and diffusive oxygen uptake (DOU). A developed quantitative model relating brGDGT methylation and isomerization degrees to DOU positions brGDGTs as an indicator for assessing organic carbon degradation and microbial diagenetic activity in deep-sea environments.
Research sample	Hadal trenches are the deepest parts of the Earth's oceans, and sampling in these areas poses significant challenges, making sediment samples from the trench regions extremely valuable and rare. Sediment samples from the Kermadec, Atacama, and Mariana trench regions were collected during three research cruises aboard the R/V Tangaroa (November to December 2017), R/V Sonne (March 2018), and R/V Zhangjian (December 2016 to Feburary 2017), respectively. This study acquired four cores from the Kermadec Trench (K3, K4, K6 and K7; water depth, 6,080 – 9,560 m; core length, 25 – 40 cm), eight cores from the Atacama Trench (A2 – 7, A9 and A10; water depth, 4,045 – 8,090 m; core length, 10 – 35 cm), and one core from the Mariana Trench (M1; water depth, 10,840 m; core length, 11 cm). All obtained samples were analyzed without any selection bias. The reason for studying these samples is that sediments in these deep-sea trench regions are characterized by extremely high pressure (> 40 MPa) and consistent bottom water temperature (ca. 2°C)36. Owing to their relatively long distance from major landmasses, their sedimentary organic carbon exhibits minimal terrestrial influence, predominantly originating from marine sources. Additionally, these sites present varied oxygen microprofiles, encompassing a diffusive oxygen uptake (DOU) range of over one order of magnitude. These characteristics make our targeted trench systems ideal for investigating the linkage between benthic oxygen conditions and marine-derived brGDGTs. Notably, each targeted trench region lies beneath water columns with varying levels of productivity, ranging from the oligotrophic Mariana, to the mesotrophic Kermadec, and to the eutrophic Atacama regions. Considering the sampling challenges in hadal trenches, the 13 short cores obtained hold significant representativeness. They provide an adequate dataset for statistical analysis of the relationship between branched glycerol dialkyl glycerol tetraethers (brGDGTs) and DOU.

Sampling strategy	The sediment cores were collected using multi-corer, box corer, and lander techniques. For sediment samples from the Kermadec Trench region, the sediment cores were sliced at 1 cm intervals for the top 2 cm, 2 cm intervals for the depth range of 2 – 10 cm, and 5 cm intervals for depths greater than 10 cm. For sediment samples from the Atacama Trench region, the sediment cores were sliced at 1 cm intervals for the top 10 cm, 2.5 cm intervals for the depth range of 10 – 20 cm, and 5 cm intervals for depths greater than 20 cm. While for sediment samples from the MT region, the sediment cores were sliced at 1 cm intervals throughout the core. This sampling approach yielded 10-16 sediment samples from each core. All samples were analyzed without any selection bias.
Data collection	All marine sediments investigated in this study were collected by authors R.N.G., F.W., and Y.X. Bacterial tetraether lipids were extracted and analyzed using an Agilent 1260 series High-Performance Liquid Chromatography (HPLC) system coupled with an Agilent 6135B quadrupole Mass Spectrometer (MS) with an Atmospheric Pressure Chemical Ionization (APCI) source. These data collection was carried out by the paper's first author, W.X.
Timing and spatial scale	Sediment samples were collected from the Kermadec, Atacama, and Mariana trench regions during three research cruises aboard the R/V Tangaroa (November to December 2017), R/V Sonne (March 2018), and R/V Zhangjian (December 2016 to Feburary 2017), respectively. Branched glycerol dialkyl glycerol tetraether lipids (brGDGTs) were extracted from the sediments in 2018-2019 at Shanghai Ocean University, Shanghai, China. The brGDGT measurements were carried out in 2020-2021 at the Southern University of Science and Technology, Shenzhen, China.
Data exclusions	We did not perform data exclusion in the analysis.
Reproducibility	Prior to the extraction of branched glycerol dialkyl glycerol tetraether lipids (brGDGTs) from all collected sediment samples, a specific quantity of C46-GTGT was introduced as an internal standard. All data have been documented in the equipment and brGDGT concentration and abundance data have been made publicly accessible.
Randomization	Randomization is not relevant in this study as all sediment samples were subjected to identical laboratory processing and data collection procedures.
Blinding	Binding is not relevant in this study. Sediment samples were gathered from the Kermadec, Atacama, and Mariana trench regions, each assigned a unique identifier and subjected to identical laboratory processing and data collection procedures.

Did the study involve field work? ☒ Yes ☐ No

Field work, collection and transport

Field conditions	Samples were collected from the Kermadec, Atacama, and Mariana trench regions during three research cruises aboard the R/V Tangaroa (November to December 2017), R/V Sonne (March 2018), and R/V Zhangjian (December 2016 to Feburary 2017), respectively. Throughout the sampling, temperatures vary between 0 to 20 degrees Celsius and are occasionally interspersed with rain. The conditions are excellent, with ample support provided.
Location	Samples were collected from the Kermadec Trench, Atacama Trench, and Mariana Trench regions, with their specific locations detailed in our Supplementary Data file.
Access & import/export	Permissions were obtained through collaboration with colleagues from the National Institute of Water and Atmospheric Research (NIWA) in New Zealand, the German Embassy in Chile, and the SONNE Coordination Office (Leitstelle). All sample collection has been fully compliant with local laws and all required permits have been secured for this study.
Disturbance	No disturbance was caused by this study.

Reporting for specific materials, systems and methods

We require information from authors about some types of materials, experimental systems and methods used in many studies. Here, indicate whether each material, system or method listed is relevant to your study. If you are not sure if a list item applies to your research, read the appropriate section before selecting a response.

Materials & experimental systems

n/a	Involved in the study	
☒	☐	Antibodies
☒	☐	Eukaryotic cell lines
☒	☐	Palaeontology and archaeology
☒	☐	Animals and other organisms
☒	☐	Clinical data
☒	☐	Dual use research of concern
☒	☐	Plants

Methods

n/a	Involved in the study	
☒	☐	ChIP-seq
☒	☐	Flow cytometry
☒	☐	MRI-based neuroimaging

Plants

Seed stocks	N/A
Novel plant genotypes	N/A
Authentication	N/A
